# Retraction: Dihydrotanshinone I inhibits hepatocellular carcinoma cells proliferation through DNA damage and EGFR pathway

**DOI:** 10.7717/peerj-15022/retraction

**Published:** 2024-01-19

**Authors:** PeerJ Editorial Office

Retraction: Wang L, Xu X, Chen D, Li C. 2023. Dihydrotanshinone I inhibits hepatocellular carcinoma cells proliferation through DNA damage and EGFR pathway. PeerJ 11:e15022 https://doi.org/10.7717/peerj.15022

The authors request that this publication be Retracted due to two significant issues which compromise the integrity of the study.

Firstly, the authors would like to acknowledge that the publication of certain data in the manuscript was not authorized by the investigators who assisted in those experiments. This was an oversight on our part and we sincerely apologize.

Secondly, researchers who should have been included as authors (due to their significant contributions) were omitted from the author list. We recognize the importance of acknowledging all contributors and their respective roles in the research and also sincerely apologize for these omissions.

PeerJ Editorial Office. 2024. Retraction: Dihydrotanshinone I inhibits hepatocellular carcinoma cells proliferation through DNA damage and EGFR pathway. PeerJ 12:e15022/retraction https://doi.org/10.7717/peerj-15022/retraction

